# *Colletotrichum fructicola*-induced fungal keratitis: a case report and literature review

**DOI:** 10.1186/s12348-026-00570-5

**Published:** 2026-04-07

**Authors:** Shanshan Ma, Xixi Gu, Mingwu Li, Jiyang Tang, Henan Li, Xiaojuan Wang, Hui Wang

**Affiliations:** 1 Department of Clinical Laboratory, Peking University People’s Hospital, Beijing, China; 2 Department of Clinical Laboratory, Caoxian People’s Hospital, Heze, Shandong China; 3 Department of Laboratory Medicine, Peking University People’s Hospital, Qingdao, Women and Children’s Hospital, QINGDAO UNIVERSITY, Qingdao, Shandong China; 4 Department of Ophthalmology, Peking University People’s Hospital, Beijing, China; 5 Beijing Key Laboratory of Innovative & Transformable Warning and Intervention Technologies for Drug-Resistant Pathogens, Beijing, China

**Keywords:** *Colletotrichum fructicola*, Fungal keratitis, ITS sequencing

## Abstract

**Background:**

Fungal keratitis, a serious corneal infection that can lead to visual impairment and blindness, is commonly caused by *Fusarium*, *Aspergillus*, and *Candida* species; however, infections caused by *Colletotrichum fructicola* (*C. fructicola*) are rare. We report the first case of *C. fructicola* keratitis diagnosed in our hospital, and review the current literature on human infections caused by this fungus.

**Case report:**

A 66-year-old male agricultural worker presented with conjunctival hyperemia in the right eye after suffering a puncture from a chestnut thorn injury. Corneal scrape samples were collected. Morphological assessment of the colonies and internal transcribed spacer (ITS) sequencing confirmed fungal keratitis caused by *C. fructicola*. After ocular debridement, the patient underwent surgical intervention to remove the foreign body from the right eye. Postoperatively, the topical antifungal voriconazole was administered. The patient subsequently showed improvement in his clinical condition. During treatment, an improvement in corneal clarity was observed, culminating in a significant enhancement of visual acuity from 0.3 to 0.6 by 3 months postoperatively.

**Conclusion:**

Although traditional morphological methods have inherent limitations in reliably identifying *C. fructicola*, their combination with ITS sequencing enables rapid and accurate species-level diagnosis. Treatment for *Colletotrichum* keratitis involves aggressive topical antifungal treatment, escalation to systemic antifungal therapy, and surgery in severe cases.

## Introduction

Fungi belonging to the genus *Colletotrichum* predominantly exhibit a plant-associated life cycle and function as either endophytes or phytopathogens that infect various plant tissues, including leaves, stems, and fruits. Notably, specific species within this genus, including *C. boninense*, *C. gloeosporioides*, and *C. fructicola*, have been identified as causative agents of human keratitis [1]. Nevertheless, clinical data on keratitis associated with *Colletotrichum* species (spp.) remain scarce, and information on the morphological identification, culture-specific traits, and efficacious treatment modalities is lacking. This paucity of information poses challenges for timely and accurate diagnosis.

Here, we describe a rare case of fungal keratitis induced by *C. fructicola* that developed after corneal trauma caused by a chestnut burr and was successfully managed. To our knowledge, this is the first case of *C. fructicola* keratitis diagnosed in our hospital and confirmed by molecular sequencing. A comprehensive review of the existing literature addressing the clinical manifestations of this condition was performed, highlighting the best diagnostic approaches to facilitate rapid identification and enable more precise therapeutic interventions in the future.

## Case review

A 66-year-old male farmer presented to our ophthalmology department for ocular treatment 4 days after sustaining an ocular injury caused by a chestnut shell during chestnut harvesting. The injury led to tearing, ocular discomfort, and a progressive decline in visual acuity to 0.3 in his right eye. The initial ophthalmic examination on admission revealed a white infiltrative lesion (approximately 5 mm in diameter) in the nasal aspect of the cornea, with an immune ring at the corneal center and suspected satellite lesions. Surgical intervention was performed on the same day of presentation, including corneal lesion debridement, removal of the deep corneal foreign body, intrastromal drug injection, conjunctival flap coverage, and subconjunctival injection. Corneal scrapings collected intraoperatively were immediately sent to the microbiology laboratory for culture analysis. Based on the clinical presentation and confocal microscopy findings, the attending physicians initiated a combined therapy of voriconazole antifungal treatment and surgical intervention, and this pharmacological regimen remained unchanged before and after the definitive etiological diagnosis.

**Fig. 1:**
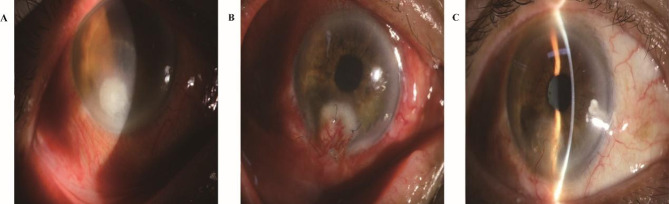
Changes in eye vision after 3 months of antifungal treatment. **A**, Initial fundus photograph of the patient upon admission, without any treatment. **B**, Fundus photograph of the patient taken 1 week after surgery. **C**, Fundus photograph of the patient taken 3 months after surgery

The patient’s clinical manifestations markedly improved, and he was discharged on postoperative day 4 in a stable condition. Following discharge, the patient continued a pharmacological regimen comprising oral itraconazole capsules along with topical administration of 1% voriconazole, tobramycin, and compound tropicamide eye drops. The patient’s visual acuity evolved from 0.3 before surgery to 0.2 one week postoperatively, which subsequently improved to 0.6 3 months postoperatively (Fig. 1).

**Fig. 2:**
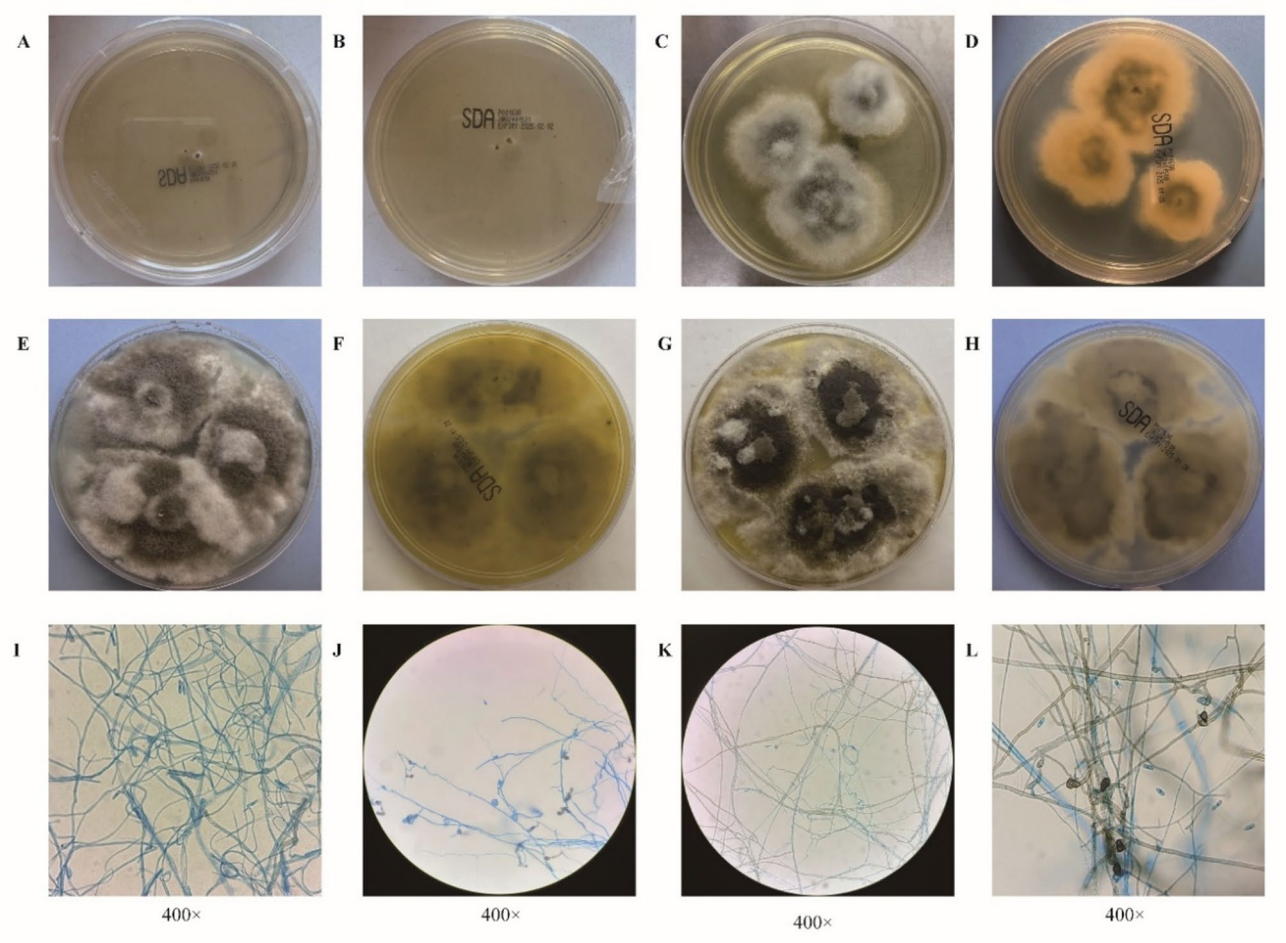
Morphological characteristics of *C. fructicola* colonies grown on SDA at 28 °C for 1, 3, 5, and 7 days, along with microscopic features after lactophenol cotton blue staining. **A**&**B**, Day 1, punctate colony growth (front and back sides). **C**&**D**, Day 3, colonies with gray-green centers and white fluffy margins; the reverse side shows central dark brown pigmentation (front and back sides). **E**&**F**, Day 5, colonies with dark gray-green centers and white, cottony peripheries, reverse exhibit dense, punctate, dark brown pigmentation (front side and back side). **G**& **H**, Day 7, colonies with gray-brown centers and broad, white, fluffy edges, abundant aerial hyphae present. Terminally swollen, darkly pigmented appressoria (front and back). **I-L**, the microscopic features following lactophenol cotton blue staining, observed on 1, 3, 5, and 7 days of grown on SDA

After 24 h of incubation at 35 °C, the corneal scraping specimen obtained from the patient yielded small white cotton-like colonies on the blood agar, indicative of fungal proliferation. These colonies were subsequently transferred and subcultured onto Sabouraud dextrose agar (SDA) for further incubation at 35 °C. By day 3, the cells had matured into slowly expanding white, fluffy, circular colonies measuring approximately 5 mm in diameter. To further assess growth characteristics, the original colonies were again inoculated onto SDA and incubated at 28 °C. At this lower temperature, the fungus demonstrated markedly accelerated growth; the initial white, fluffy colonies expanded into circular colonies approximately 25 mm in diameter by day 3, exhibiting a gray-green central region with a white, cottony periphery. The reverse surface of the colony exhibited dark brown pigmentation that gradually intensified over time and culminated in the formation of dense, punctate dark brown spots. Colonies cultured for 2, 3, and 7 days were stained with lactophenol cotton blue and examined under a microscope using the cellophane tape method. High-power magnification microscopy revealed septate hyphae, capsule-like conidia arranged in parallel arrays, and darkly pigmented, terminally swollen appressoria (Fig. 2). Based on these macroscopic and microscopic morphological characteristics, the isolate was provisionally identified as belonging to the genus *Colletotrichum*. Colonies incubated for 2 days on SDA were further selected for identification using both BioMérieux (Marcy-l’Etoile, France) and Bruker Rapiflex MALDI-TOF/TOF mass spectrometry systems (Bruker Daltonics, Bremen, Germany). MALDI-TOF MS was used for the preliminary identification of the fungal isolate, yet no definitive identification at the genus or species level was achieved via database matching. For the purpose of molecular identification, genomic DNA was extracted from the fungal mycelium by following a protocol that adhere to the instructions specified in the commercial kit (E.Z.N.A.^®^ HP Fungal DNA Kit). The internal transcribed spacer (ITS) region of rDNA was amplified with the universal primer pair ITS1/ITS4 [2] and sequenced via Sanger sequencing at RuiBio BioTech (Beijing). The resulting sequence was aligned in the NCBI database (https://blast.ncbi.nlm.nih.gov/Blast.cgi), showing 100% identity with *C. fructicola* (MF554831.1).

In vitro antifungal susceptibility testing was subsequently performed using a colorimetric microdilution assay (YeastOne BioMérieux, Marcy-l’Étoile, France), with the inoculum suspension prepared in accordance with the guidelines specified for *Aspergillu*s species (spp.). Given the absence of the breakpoints for *C. fructicola*, the minimum inhibitory concentrations (MICs) were ascertained by observing colorimetric changes, following the commercialization specifications provided for *Aspergillus* species (spp.). These findings revealed a comparatively elevated MIC for fluconazole, whereas other antifungal agents, such as voriconazole, itraconazole, posaconazole, 5-fluorocytosine, amphotericin B, and the echinocandin class of drugs, all exhibited low MICs, indicating superior in vitro efficacy (Table 1).

**Table 1:** MICs or minimum effective concentrations (MECs) of antifungal agents against *Colletotrichum* isolates from fungal keratitis cases

Fungal species	Antifungal susceptibility testing method	MIC or MEC (mg/L)											Reference
		AMB	ITC	VRC	POS	5 FC	KTC	MCC	AFG	CAS	FLC	MFG	
*C. truncatum*	NM	0.5	2	16	0.5	>64	2	2					[3]
*C. gloeosporioides*	Broth microdilution (Sensititre, YeastOne )	0.25	0.25	0.25	0.25	16			0.12	0.25	64	0.06	[4]
*C. gloeosporioides*	NM	0.5	8	0.5					0.5	0.5	>64	0.25	[5]
*C. dematium*	Broth microdilution (Sensititre, YeastOne)	0.125	1	1	1	32			0.125	0.125	128	0.03	[6]
*C. chlirophyti*	Broth.microdilution (Standard methods)	0.125	4	2	1								[7]
*C. truncatum*	NM	0.125		0.25	0.25							0.25	[8]
*C. fructicola*	Broth.microdilution (Sensititre, YeastOne )	2	0.25	1	0.25	1			0.12	0.25	>256	0.12	This study

Note: AMB, amphotericin B. ITC, itraconazole. VRC, voriconazole. POS, posaconazole. 5 FC, 5 flucytosine. KTC, ketoconazole. MCC, miconazole. AFG, anidulafungin. CAS, caspofungin. FLC, fluconazole. MFG, micafungin. NM, not mentioned. Sensititre YeastOne, Sensititre^®^ YeastOne Susceptibility plates (from the Thermo Fisher Scientific, Waltham, MA or from the Trek Diagnostic System Ltd., East Grinstead, UK). In the standard broth microdilution method, MIC for amphotericin B, itraconazole, posaconazole, and voriconazole is the lowest concentration with 100% growth inhibition, while MEC for caspofungin and micafungin is the lowest concentration causing morphologic changes

At the 2-week follow-up assessment, the patient exhibited a notable reduction in corneal infiltration, with visual acuity in the right eye improving to 0.4. During postoperative week 3, the systemic therapeutic regimen was modified by adjusting the dose of oral itraconazole to 0.2 g once daily. By week 8 of follow-up, the infection had resolved satisfactorily, prompting the discontinuation of antifungal therapy, and the patient was maintained under close observation.

**Fig. 3:**
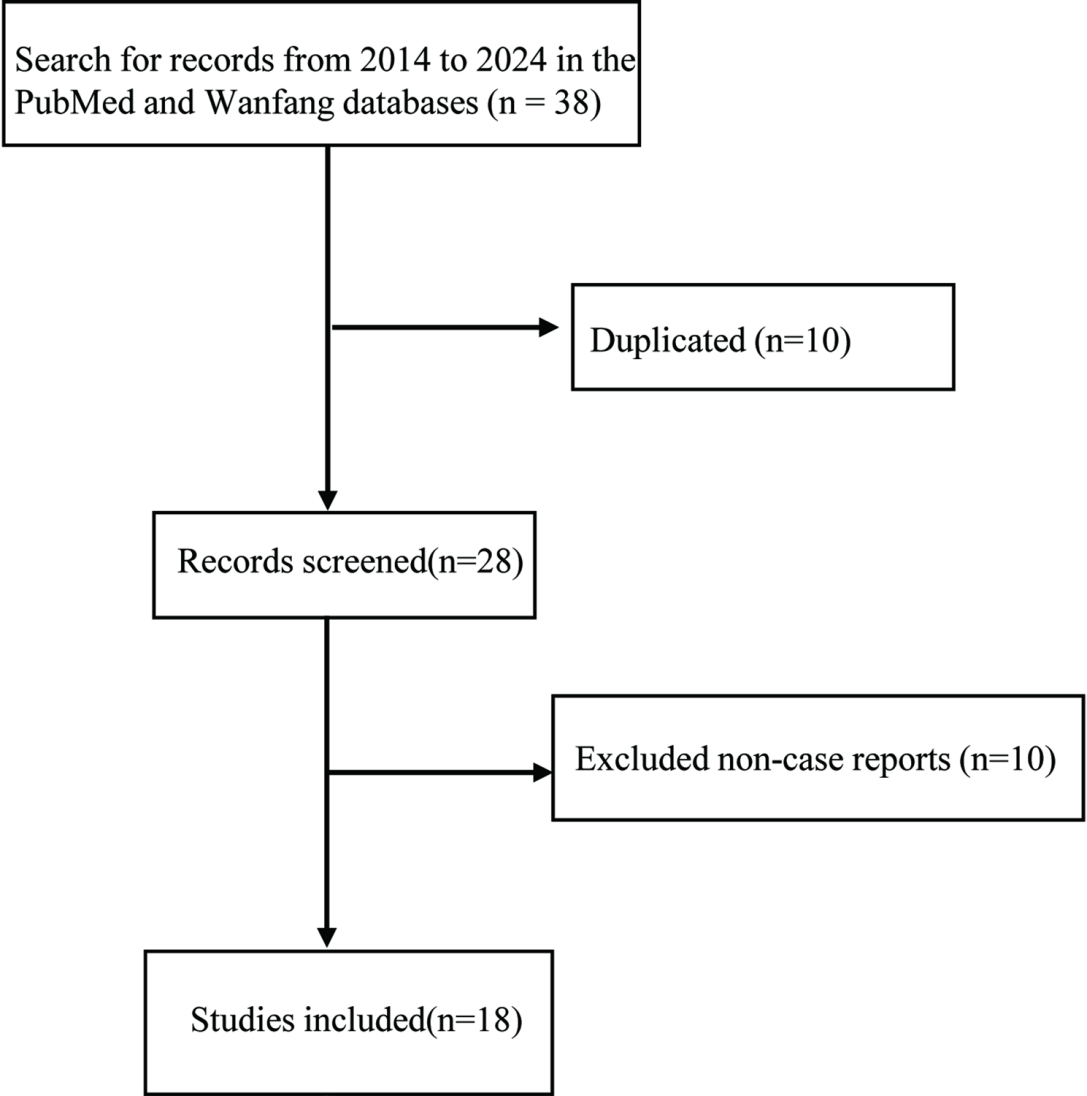
Flow diagram for the scoping review process

A systematic literature search of the PubMed database using the key search term “*Colletotrichum* keratitis” retrieved 24 articles published between 2014 and 2024. Of these, 17 were case reports that provided detailed accounts of clinical manifestations and treatment outcomes. Moreover, an additional case report sourced from the Wanfang Database during the same timeframe offered comprehensive diagnostic and therapeutic insights. In total, 18 case reports were thoroughly reviewed (Fig. 3), and pertinent data were extracted for comparative analysis (Table 2).

**Table 2:** A literature review of *Colletotrichum* keratitis

Country/year	Age/Sex	Occupation	Risk factor	Ocular Features	Clinical Symptoms	Diagnostic Method	Identification Result	Empirical Treatment	Targeted Therapy	Outcome	Reference
India/2015	54/M	Electrician	No trauma	Corneal ulcer, stromal infiltration, no feathery margins	Ocular pain, mild watering, photophobia, gradual loss of vision in right eye	Morphology	*C. coccodes*	Topical norfloxacin eye drops	Ocular administration of amphotericin B for 2 months	Clinical cured: complete healing of the ulcer and restoration of normal vision	18
Iran/2018	56/M	Farmer	Minimal ocular trauma with rice plant	Conjunctival injection, corneal infiltration in infranasal quadrant with gritty appearance, feathery border	Ocular pain and burning	Morphology	*C. coccodes*	Topical natamycin q1h, levofloxacin q4h, lubricating agents	2 weeks for systemic anti-fungal therapy, and topical natamycin tapered weekly until 6th week when developed a scar	Clinical improvement	19
Canada/2015	87/M	NM	No ocular trauma or contact lens wear	Conjunctival congestion, hypopyon	Progressively increasing pain, redness, excessive tearing, decreased vision, and lid swelling in left eye	Pathological, Morphology, DNA sequencing of ITS2, D1/D2, beta-tubulin genes	*C. truncatum* species complex	Topical moxifloxacin for 3 weeks, the prednisolone drops q2h, 5% atropine tid, Urgent pars plana vitrectomy and lensectomy + PKP	0.15% amphotericin B, subconjunctival depot	Failure: evisceration	12
China/2024	54/F	NM	Cornea scratched by grass	Feathery stromal infiltration, approximately 60% depth of the cornea	Eye redness and discomfort, blurred vision	Morphology, MALDI-TOF MS, NGS	*C. truncatum*	NM	LKP, 1% voriconazole eye drops qid for 2 weeks	Clinical improvement	1
	51/F	NM	Cornea scratched by a twig	Feathery stromal infiltration, approximately 60% depth of the cornea	Eye redness and discomfort, blurred vision	Morphology, MALDI-TOF MS, NGS	*C. truncatum*	NM	LKP, 1% voriconazole eye drops qid for 2 weeks	Clinical improvement	
	66/F	NM	Cornea scratched by grass	Corneal ulcer with total depth stromal infiltration and endothelial plaque	Eye redness and discomfort, blurred vision	Morphology, MALDI-TOF MS, NGS	*C. truncatum*	NM	PKP, 1% voriconazole eye drops qid for 2 weeks	Clinical improvement	
	46/M	NM	Unknown	Corneal ulcer with total depth stromal infiltration and endothelial plaque	Eye redness and discomfort, blurred vision	Morphology, MALDI-TOF MS, NGS	*C. gloeosporioides*	NM	PKP, 1% voriconazole eye drops qid for 2 weeks	Clinical improvement	
	57/M	NM	Mosquito flew into eye	Corneal ulcer with total depth stromal infiltration and endothelial plaque	Eye redness and discomfort, blurred vision	Morphology, MALDI-TOF MS, NGS	*C. gloeosporioides*	NM	PKP, 1% voriconazole eye drops qid for 2 weeks	Clinical improvement	
	77/M	NM	Cornea scratched by a twig	Corneal ulcer with total depth stromal infiltration and endothelial plaque	Eye redness and discomfort, blurred vision	Morphology, MALDI-TOF MS, NGS	*C. gloeosporioides*	NM	PKP, 1% voriconazole eye drops qid for 2 weeks	Clinical improvement	
	55/M	NM	Corneal injury by chestnut burrs	Corneal ulcer with total depth stromal infiltration and endothelial plaque	Eye redness and discomfort, blurred vision	Morphology, MALDI-TOF MS, NGS	*C. fructicola*	NM	PKP, 1% voriconazole eye drops qid for 2 weeks	Clinical improvement	
	59/F	NM	Corneal injury by chestnut burrs	Corneal ulcer with total depth stromal infiltration and endothelial plaque, hypopyon in the anterior chamber	Eye redness and discomfort, blurred vision	Morphology, MALDI-TOF MS, NGS	*C. fructicola*	NM	PKP, 1% voriconazole eye drops qid for 2 weeks	Failure: evisceration	
	54/M	NM	Corneal injury by chestnut burrs	Corneal ulcer with total depth stromal infiltration and endothelial plaque	Eye redness and discomfort, blurred vision	Morphology, MALDI-TOF MS, NGS	*C. fructicola*	NM	PKP, 1% voriconazole eye drops qid for 2 weeks	Clinical improvement	
India/2020	12/M	NM	Thorn injury	Anterior stromal corneal infiltration with stromal thinning and satellite lesions	Eye pain and redness	Morphology, ITS sequencing	*C. truncatum*	Topical 5% natamycin eye drops q1h + 1% itraconazole bid + gatifloxacin six times daily	Topical 5% natamycin q1h + 1% voriconazole e/d	Clinical improvement	20
China/2020	52/M	Farmer	Eye injured by an apple tree branch	Corneal ulcer, peripheral corneal edema, anterior chamber abscess	Eye progressive pain and redness, excessive tearing, diminution of vision	Morphology, MALDI-TOF MS, ITS sequencing	*C. gloeosporioides*	Topical levofloxacin and tobramycin for 8 days	Surgery, following topical 0.05% amphotericin B ointment, oral voriconazole (200 mg×2 tablets)	Clinical improvement	13
Spain/2019	45/M	NM	Eye trauma by orange tree branch	Corneal abscess, conjunctival hyperemia, hypopyon	Eye pain, redness	Morphology, DNA sequencing	*C. gloeosporioides*	Doxycycline, moxifloxacin, ceftazidime, vancomycin	Oral voriconazole 200 mg q12h for 2 month+ 1% voriconazole eye drops q2h + anterior chamber irrigation	Loss to follow-up	21
Australia/2022	71/M	NM	No obvious trauma history	Corneal stromal infiltration, hypopyon	Eye discomfort	Morphology, DNA sequencing	*Glomerella cingulata* (a variant of *C. gloeosporioides*)	Topical 1% ofloxacin + oral doxycycline 100 mg qd, intravitreal (ceftazidime + vancomycin + voriconazole, oral voriconazole) + oral voriconazole 400 mg q12h	Intravitreal amphotericin B (0.005 mg/0.1 mL), fortnightly for 6 weeks, systemic voriconazole continued.	Failure: evisceration	22
Iran/2021	69/M	Farmer	Right eye injured by herbal material	Superficial corneal infiltration without hypopyon	Vision loss	Morphology, ITS sequencing	*C. gloeosporioides*	Topical 1% voriconazole + vancomycin + ceftazidime hourly	Voriconazole every hour + levofloxacin q4h for 2 months	Clinical improvement	14
Cuba/2016	41/M	Pig farmer	No ocular trauma	Mild eye edema, conjunctival congestion, yellow corneal infiltration	Burning ocular pain, blurred vision, redness of left eye	Morphology, DNA sequencing of ITS2, D1/ D2 region, and beta-tubulin locus genes	*C. truncatum*	Topical 3% ceftazidime + 3% vancomycin, one eye drops every hour	1% miconazole one eye drop every hour, 1%ketoconazole one eye drops tid, and oral ketoconazole 200 mg qd for 7 days); PKP, topical 5% natamycin eye drops every 30 min	Clinical improvement	24
	70/M	NM	No ocular trauma	Corneal defect with central diffuse infiltration, hypopyon	Vision loss, eye discomfort and secretions in right eye	Morphology, DNA sequencing of ITS2, D1/ D2 region, and beta-tubulin locus genes	*C. truncatum*	Topical 3% gentamicin q4h for 2 days; 5% ceftazidime + 3% vancomycin eye drops every 30 min for 5 days.	1% miconazole + 5% natamycin + 5% moxifloxacin, one eye drop every hour for 7 days; PKP, topical natamycin for 2 weeks	Clinical improvement	
India/2022	34/M	Pig farmer	Foreign body fell in the eye during farming	Corneal ulcer	Eye pain, redness	Morphology, molecular sequencing	*C. asianum*	NM	Topical 5% natamycin + 1% voriconazole drops hourly/2 hourly for 24 h/another 24 h, repeat 6 times; topical natamycin continued qid for 2 weeks	Clinical improvement	23
	60/M	Farmer	Ocular trauma with vegetative material	Corneal ulcer, corneal stromal infiltration	Eye pain, redness	Morphology, molecular sequencing	*C. asianum*	NM	Topical 5% natamycin + voriconazole (1%) eye drops hourly/2 hourly for 24 h/another 24 h, repeat 6 times; oral ketoconazole 400 mg qd for 1-week, topical natamycin qid for the next 2 weeks	Clinical improvement	
Czech Republic/2019	56/F	NM	Poked with eyeliner	Paracentral corneal ulcer without intraocular inflammation	Eye pain, tearing	Microscopy, DNA sequencing of ITS, TUB2 and GAPDH genes	*C. dematium*	Topical tobramycin drops once an hour + levofloxacin drops daily	Topical and systemic amphotericin B, PKP, oral itraconazole	Clinical improvement	15
USA/2021	82/M	NM	Post-keratoplasty, no trauma	Corneal and retinal infiltrative lesion	Ocular discomfort	Morphology, DNA sequencing of ITS, BenA and GenE	*C. chlorophyti*	Voriconazole intravitreal injections (50mcg/0.1mL) for a total six doses, topical and oral voriconazole (200 mg q12h) for 8 days	Intravitreal amphotericin B injection (5mcg/0.1 ml) for a total four doses, repeat corneal transplant	Failure: evisceration	16
China/2020	62/M	Farmer	Twig injury	Corneal ulcer	Eye pain, decreased visual acuity	Morphology, DNA sequencing of ITS, ACT, TUB2, CHS1 and GAPDH genes	*C. truncatum*	0.5% levofloxacin	No antifungal drugs used	Failure: evisceration	3
	76/M	NM	No trauma	Corneal ulcer with feathery stromal infiltration	Eye pain, decreased visual acuity	Morphology, DNA sequencing of ITS, ACT, TUB2, CHS2 and GAPDH genes	*C. fusiforme*	0.5% levofloxacin	Oral fluconazole + voriconazole	Failure: evisceration	
	72/M	Farmer	Dirt water contact	Corneal ulcer with feathery stromal infiltration, minimal hypopyon	Eye pain, decreased visual acuity	Morphology, DNA sequencing of ITS, ACT, TUB2, CHS3 and GAPDH genes	*C. tropicale*	0.5% levofloxacin	Topical natamycin	Clinical improvement	
	22/M	NM	Injured by metallic foreign body	Corneal ulcer with feathery stromal infiltration	Eye pain, decreased visual acuity	Morphology, DNA sequencing of ITS, ACT, TUB2, CHS4 and GAPDH genes	*C. tropicale*	0.5% levofloxacin	No antifungal drugs used	Clinical improvement	
	79/F	Farmer	Soil contact	Corneal ulcer with feathery stromal infiltration	Eye pain, decreased visual acuity	Morphology, DNA sequencing of ITS, ACT, TUB2, CHS5 and GAPDH genes	*C. fructicola*	0.5% levofloxacin	Topical amphotericin B + voriconazole	Clinical improvement	
	63/F	Farmer	Twig injury	Corneal ulcer with feathery stromal infiltration	Eye pain, decreased visual acuity	Morphology, DNA sequencing of ITS, ACT, TUB2, CHS6 and GAPDH genes	*C. tropicale*	0.5% levofloxacin	No antifungal drugs used	Clinical improvement	
	77/F	NM	Unknown	Corneal ulcer with feathery stromal infiltration	Eye pain, decreased visual acuity	Morphology, DNA sequencing of ITS, ACT, TUB2, CHS7 and GAPDH genes	*C. fructicola*	0.5% levofloxacin	Topical natamycin and amphotericin B	Clinical improvement	
China/2020	43/F	Farmer	Eye scratched by wheat husk	Corneal infiltration to deep stroma, satellite lesions, feathery margins	Redness in left eye, blurred vision and corneal opacity	Morphology, ITS sequencing	*C. truncatum*	Topical levofloxacin	Topical 0.3% tobramycin ointment + 0.3% gatifloxacin gel, oral terbinafine hydrochloride 0.25 g/tablet	Failure: evisceration	25
Spain/2016	56/F	Farmer	Corneal injury by an orange tree branch	Conjunctival hyperemia without secretion, corneal ulcer, hypopyon	Vision loss	Conventional culture and PCR	*C. gloeosporioides*	Moxifloxacine 5 mg/mL eye drops + oral moxifloxacin 400 mg qd	PKP, prednisolone acetate 10 mg/1 ml q4h, moxifloxacin 5 mg/ml 5 times a day and amphotericin B 0.5 mg/ml 5 times a day, voriconazole 200 mg qd	Clinical improvement	26
India/2017	45/M	Farmer	Ocular trauma by stone chip	Central corneal ulcer, feathery edge in anterior stroma, no satellite lesions and no hypopyon	Eye progressive pain, excessive tearing, redness in right eye, blurring of vision	Morphology, ITS1/4 sequencing	*C. gloeosporioides*	Topical gentamicin eye drops	Topical 0.15% amphotericin B every 30 min/1 hour for 24 h/another 24 h, q12h for 10 days; plus oral voriconazole (400 mg q12h for 2 days, then 200 mg q12h for 10 days; then topical 5% natamycin	Clinical cured: complete healing of the ulcer and restoration of normal vision	27
Spain/2016	75/M	Farmer	Ocular trauma by orange tree branch	Corneal edema, high intraocular pressure; corneal ulcer, hypopyon	Vision loss	Morphology, molecular sequencing	*C. gloeosporioides*	Moxifloxacin 5 mg/mL qid + ciprofloxacin 3 mg/g at night for 4 weeks	Oral 400 mg q12h loading dose and 200 mg q12 h maintenance dose, topical 10 mg/mL daily for 7 weeks, and intravitreal (100 mcg/0.1 mL), and intrastromal 1% voriconazole treatment, PKP	Failure: evisceration	28

Abbreviations: NM, not mentioned. ICVM, in vivo confocal microscopy. NGS, next-generation sequencing. LKP, lamellar keratoplasty. PKP, penetrating keratoplasty. q1h, once every hour. qid, four times daily. M, male. F, female. MALDI-TOF MS, matrix-assisted laser desorption/ionization-time-of-flight mass spectrometry. NGS, next-generation sequencing

## Discussion

We conducted a systematic review, highlighting the emerging role of *Colletotrichum* species (spp.) in ocular infections over the past decade. *Colletotrichum* species (spp.) are increasingly recognized as causative agents of fungal keratitis, as evidenced by regional reports from China, and South Korea. Studies indicate a rising proportion of fungal keratitis cases attributed to *Colletotrichum* species (spp.), reaching 6.6–11.1% in recent years, a trend likely reflecting climate change and global warming, improved diagnostics and greater clinical awareness [11–13]. Beyond keratitis, these fungi have been implicated in subcutaneous and deep soft-tissue infections, often following plant-related trauma, particularly in immunocompromised individuals. Such reports highlight a broadening clinical spectrum that may involve multiple organ systems, posing diagnostic and therapeutic challenges.

This study presents a case of fungal keratitis caused by *C. fructicola*. Patients typically exhibit conjunctival hyperemia, tearing, and blurred vision; however, the initially mild and indolent course often leads to diagnostic delay and inappropriate empirical antibacterial therapy. Corneal ulcers with feathery edges, satellite lesions, or hypopyon provide key diagnostic clues. Accurate diagnosis integrates clinical, morphological, and molecular approaches. While conventional morphology permits genus-level recognition, differentiation of closely related species requires molecular methods, notably internal transcribed spacer (ITS) sequencing, which significantly enhances diagnostic precision [16].

Here, we further analyze and refine the diagnostic techniques and therapeutic strategies for fungal keratitis, building on a previous review by Kaliamurthy J et al. on keratitis caused by *Coelomycetous fungi*, whose diagnosis and treatment options are currently limited [21]. The advancement and integration of diagnostic technologies are crucial for addressing rare fungal keratitis. Although traditional microscopy and culture serve as foundational methods, their sensitivity and timeliness are limited. While matrix-assisted laser desorption/ionization time-of-flight mass spectrometry (MALDI-TOF MS) has become widely adopted, its databases often lack reference spectra for rare molds, particularly phytopathogens, frequently preventing species-level identification. Therefore, for such pathogens, sequencing of the ITS region becomes an essential confirmatory tool due to its broader database coverage and higher resolution [22]. We recommend a stepwise, integrated diagnostic pathway: initial screening with microscopy and culture, followed by MALDI-TOF MS for common agents, and reflexive ITS sequencing for isolates that are unidentifiable or suspected to be rare, to achieve a precise diagnosis [23]. In managing these severe infections, therapeutic strategies must also evolve. Emerging host-directed approaches, such as monoclonal antibodies targeting conserved virulence factors like poly-N-acetylglucosamine (PNAG), have demonstrated potential as an adjunctive therapeutic strategy in experimental models, offering new directions for the future management of refractory cases [24].

Similar to other fungal infections, such as those caused by *M. phaseolina* [25] or other dematiaceous fungi [26], the treatment of *C. fructicola* infection remains a clinical challenge. Management of *Colletotrichum* keratitis typically involves combined pharmacological and surgical strategies. In a review of the retrieved reported cases (2014–2024), favorable outcomes were achieved in 78.5% of patients treated with surgery plus antifungals and 78.9% with topical antifungals alone. Voriconazole and natamycin—as monotherapy or combined with amphotericin B—were the most frequently used and effective regimens. Antifungal susceptibility varies considerably among species and regions, typically showing high MICs to fluconazole and flucytosine, and lower MICs to amphotericin B, voriconazole, itraconazole, and echinocandins. These findings underscore the importance of individualized susceptibility testing to guide therapy. In the present case, topical voriconazole following surgical intervention led to improved corneal clarity and visual acuity from 0.3 to 0.6 within three months. While triazoles such as voriconazole and itraconazole demonstrate good activity against most *Colletotrichum* isolates, clinical evidence for other agents like posaconazole or echinocandins in keratitis remains limited. Echinocandins, in particular, are not standard in filamentous fungal keratitis due to variable corneal penetration and heterogeneous expression of the drug target. Thus, extended antifungal susceptibility testing is valuable in complex or refractory cases.

Surgical interventions—such as debridement, lamellar keratoplasty (LKP), or penetrating keratoplasty (PKP)—are often required in advanced or refractory cases [27, 28]. Early removal of infected tissue not only enhances antifungal penetration but also improves clinical outcomes, especially when deep stromal layers are involved. In the present case, immediate surgical extraction of the foreign body, combined with topical and systemic voriconazole and itraconazole, resulted in marked visual recovery, highlighting the efficacy of a coordinated multimodal approach.

Given the association of *Colletotrichum* infections with plant-related trauma, clinical suspicion should be heightened in patients with agricultural exposures or injuries involving plant material. For high-risk groups such as agricultural workers, consistent use of protective eyewear is strongly recommended, along with routine ophthalmologic evaluations to enable early detection and management of post-traumatic complications.

## Conclusion

In summary, although *Colletotrichum* keratitis is uncommon, its detection has risen with advances in diagnostic methods and clinical awareness. Accurate identification relies on a multimodal strategy that integrates clinical evaluation, morphological assessment, and molecular analysis. Effective management depends on early initiation of antifungal therapy, preferably guided by susceptibility testing, along with timely surgical intervention when necessary. Further studies are required to clarify the epidemiology, virulence determinants, and optimal treatment regimens for *Colletotrichum* infections, with the overarching aim of preserving visual function and reducing the risk of blindness.

## Data Availability

Not applicable.

## Abbreviations

M: Male
F: Female
NM: Not mentioned
ICVM: In vivo confocal microscopy
NGS: Next-generation sequencing
LKP: Lamellar keratoplasty
PKP: Penetrating keratoplasty
q1h: Once every hour
bid: Twice daily
qid: Four times daily
